# The effects of prepubertal epididymal ligation upon the rat testis

**Published:** 2014-10

**Authors:** Fatih Mehmet Gur, Sema Timurkaan

**Affiliations:** 1 *Sabiha Gokcen Airport Veterinary Border Inspection Post, Ministry of Food, Agriculture and Livestock, 34912 Pendik, Istanbul, Turkey.*; 2 *Department of Histology-Embryology, Faculty of Veterinary Medicine, University of Firat, 23100 Elazığ, Turkey.*

**Keywords:** *Epididymis*, *Ligation*, *Degeneration*, *Testis*, *Rat*

## Abstract

**Background:** Previous researches about the effects of epididymal obstruction on the testes are contradictory, and the mechanism harmful effect of male duct system obstruction on seminiferous tubules still remains unclear.

**Objective:** The aim of this study was to investigate the effects of epididymal obstruction in prepubertal rats on the testis.

**Materials and Methods:** 15 days of age, the young rats were divided at random in two groups for epididymal ligation (n=25) or sham operation (n=15). Both groups were sacrificed at 21, 35, 56, 90, 120 days. The testis were removed, fixed in Bouin’s fixative and embedded in paraffin wax. The tissues were sectioned at 5 µm and stained with haematoxylin-eosin and triple stain.

**Results:** In ligated rats the first histological alterations were detected at 56 days. These degenerative changes included increase at the seminiferous tubule diameter and basal membrane thickness, decrease at the germinal epithelium thickness, depletion of spermatids and presence of multinucleated spermatids. In 90 and 120 days ligation groups; germ cells greatly reduced in number.

**Conclusion:** progressive degenerative alterations occurred in the seminiferous tubules after prepubertal epididymal obstruction but these degenerative alterations are not observed until puberta and in the seminiferous tubules that showed extensive degeneration, seminiferous epithelium was composed mainly of Sertoli cells.

## Introduction

Deleterious changes can occur in the seminiferous tubules as a result of obstruction of the male duct system. These changes are variable according to blocked region (1-3). Studies on the obstruction of male duct system are especially focused on the ductus deferens. They mainly aim to examine the effects of vasectomies, which are performed to avoid pregnancy. The results of these studies vary. In some studies, a vasectomy is claimed to cause a rapid and widespread degeneration of seminiferous epithelium, through depletion of the germinal cells completely or partially, in some or all of the seminiferous tubules (4-11). 

Some others report that vasectomies have no negative effects on the testes (3, 12). On the contrary, there are a few studies investigating the effects of epididymal obstruction, which is formed in the prepubertal period. Some studies show that epididymal obstruction leads to pathological changes in the seminiferous tubules, but some of these changes are reversible; whereas some other studies report that after partial epididymectomy, the pathological changes in the seminiferous tubules are definitive (3, 11). In studies concerning the prepubertal period, in the seminiferous tubules, serious and irreversible changes are encountered that are characterized by depletion of the germinal cells completely or partially, in the seminiferous tubules (1, 13).

The epididymis has the functions of absorbing most of the fluid leaving the testis, and of maturing and storing the spermatozoa until ejaculation. However, it has also been suggested that the epididymis may have endocrine functions (11). Therefore the studies that examined the effects of epididymal obstruction upon the testes and spermatogenesis have become more important. In the prepubertal period, there are several factors, which can cause epididymal obstruction: such as iatrogenic ties due to inguinal herniotomies, inflammatory, tumoral, cystic, and similar causes; and diseases such as the congenital absence of the vas deferens (CAVD). The investigation of the effect of these conditions on the testes, and therefore on the process of spermatogenesis, is of major importance. The purpose of this study is investigate that the effects of the above mentioned conditions on the testes and male fertility.

## Materials and methods

This experimental study was conducted on Experimental Research Unit of Firat University (Elazig). Wistar rat pups (forty in number) were obtained from at 12 days of age. The animals were kept in an air-conditioned room with a 12 hr light/dark cycle and given commercial food pellet and water *ad libitum*. When the pups reached 15 days of age, they were divided at random into groups for epididymal ligation or sham operation. Experimental procedures were approved by Firat University Local Ethics committee on Animal Experimentation.

**Surgical procedures and fixation**

All 15-days old rats were anaesthetized by an intraperitoneal injection with 50 mg/kg ketamine under local antiseptic conditions. In the ligation group, after small vertical midline abdominal incisions, the testis and epididymes were bilaterally exposed, then the each epididymis, approximately at the midpoint of the corpus, was ligated with 6-0 silk. The same incision and procedure was performed in the sham group except for the ligation of the epididymides. Pups were observed at weekly intervals postoperatively to ensure that the testes descended normally. 

Five rats in the epididymal ligation group and three sham-operated rats were killed at 21, 35, 56, 90, and 120 days. Before being killed all male pups were anaesthetized with ethyl ether. Testes were taken out rapidly and fixed in Bouin’s fixative for 36 h at 4^o^C and then dehydrated in ethanol, cleared in xylene, and embedded in paraffin wax. The tissues were sectioned at 5 µm and stained with haematoxylin-eosin and triple stain. The diameters of the seminiferous tubules and the thickness of the germinal epithelium were measured in randomly selected histological slides from the ligated and the sham operated groups. In the selected histological slide, 40 tubules were examined in different areas using an ocular micrometer at a magnification of 400X.

The histopathological changes, such as a decrease in the average diameters of the seminiferous tubules and in the thickness of the germinal epithelium, a decrease in the number of the germinal cells, varying degrees of absence of the germinal cells, presence of multinucleated spermatids, chromatin condensation in the nuclei of the spermatids, and thickening of the basal membrane were noted as signs of a degenerated tubulus.

## Results

**Figure 1:**
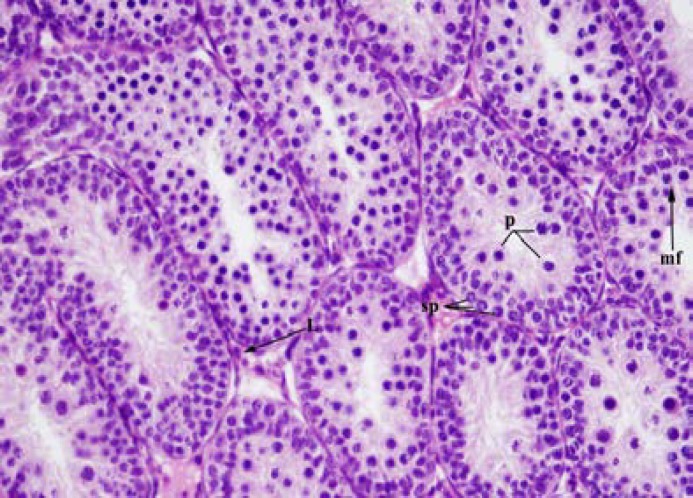
The testis of 21-day-old ligation group. p= primary spermatocytes, sp= spermatogonia, L= Leydig cell, mf= mitotic figures. Hematoxylin and eosin X 400

**Figure 2:**
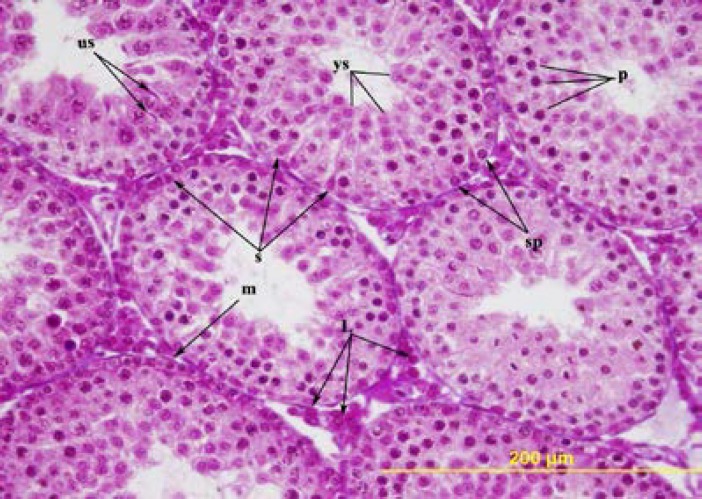
The testis of 35-day-old ligation group. m= peritubular myoid cell, p= primary spermatocytes, s= Sertoli cell, us= elongated spermatid, ys= round spermatid, sp= spermatogonia, L= Leydig cell. Triple stain X 400

No difference was observed among the testes of the rats in the group with ligation on the 21^st^ day, on the 35^th^ day, and the sham group. In the rats in the group with ligation on the 21^st^ day, in the seminiferous tubules, a number of immature Sertoli cells with oval nuclei, a few spermatogonia with round nuclei, primary spermatocytes, and mitotic figures were observed (Figure 1). In the rats in the group with ligation, on the 35^th^ day, next to the primary spermatocytes, many round spermatides near the lumen of the tubuli and elongated spermatids in some tubules were seen (Figure 2).

**Figure 3:**
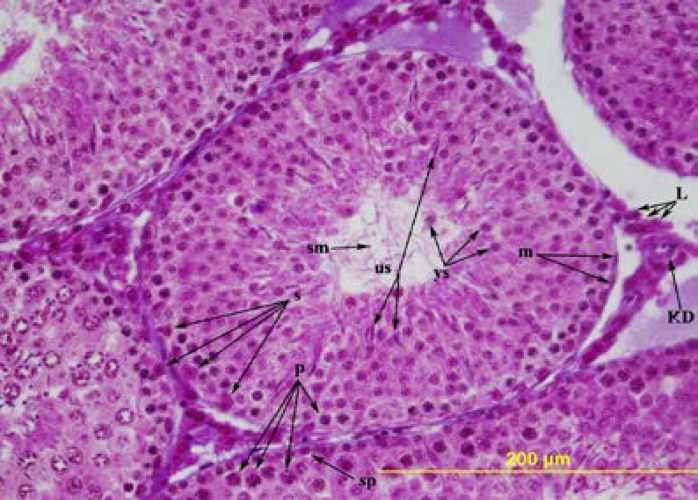
The testis of 56-day-old shame operation group. m= peritubular myoid cell, sp= spermatogonia, p= primary spermatocytes, s= Sertoli cell, ys= round spermatid, us= elongated spermatid, sm= spermium, L= Leydig cell, KD= blood vessel. Triple stain X 400

The first difference between the group with ligation and the sham group were observed on the 56^th^ day. Morphologically, the testes of the rats of the groups with ligation were larger and harder compared to the sham group. In the testes of the sham group, in the germinal epithelium, Sertoli cells, spermatogonia, spermatocytes, a considerable number of round and elongated spermatids and spermium in the tubulus lumen were present (Figure 3). Of the four rats in the groups with ligation, the average diameters of the seminiferous tubules were slightly increased, and the thickness of the germinal epithelium was decreased compared to the sham group. 

**Figure 4:**
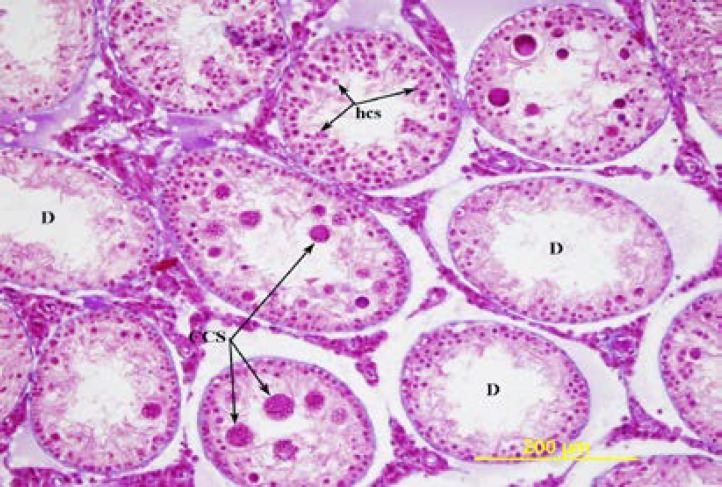
The testis of 56-day-old ligation group. D= degenerated tubules, CCS= multinucleated spermatid, hcs= crescent-nuclear spermatids. Triple stain X 200.

**Figure 5:**
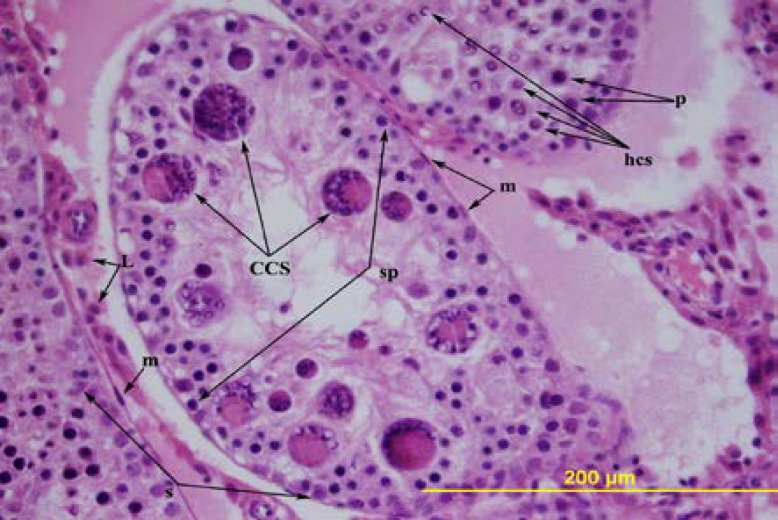
The testis of 56-day-old ligation group. M= peritubular myoid cell, sp= spermatogonia, p= primary spermatocytes, s= Sertoli cell, L= Leydig cell, hcs= crescent-nuclear spermatids, CCS= multinucleated spermatid. Hematoxylin and eosin X 400.

Although the lumens of the seminiferous tubules were wider in the ligated group, all spermatogenic cells were present in the seminiferous epithelium. In the contralateral rat testes, the thickness of the germinal epithelium was decreased. The tubuli in which the thickness was severely decreased, near the basal compartment, germinal cells with dark-stained round nuclei are observed. In these tubuli, the ad luminal compartment only consisted of cytoplasmic extensions of the Sertoli cells. In some of the tubuli, in the germinal epithelium, near the lumen, there were large multinucleate cells. The microscopic view of these cells and the nuclei of the round spermatids in some of the tubuli had a crescent or ring-shape (Figure 4, 5). 

**Figure 6:**
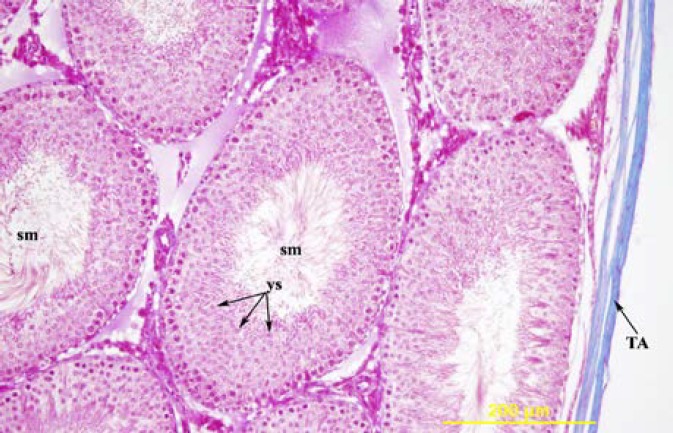
The testis of 90-day-old shame operation group. ys= round spermatid, sm= spermium, TA= tunika albuginea. Triple stain X 200.

**Figure 7:**
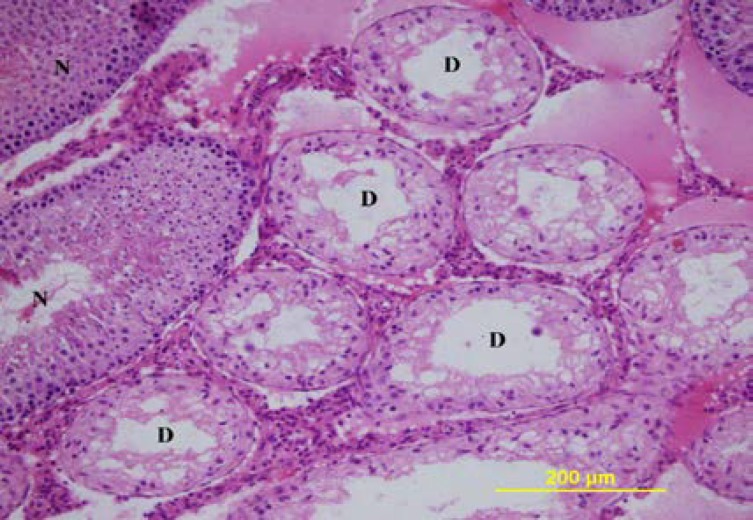
The testis of 90 day-old-ligation group. D= degenerated tubules, N= normal tubules. Hematoxylin and eosin X 200

In the testes with pathological changes by triple staining, the staining around the tubuli was more intense (Figure 4). The morphology of the Leydig cells in both groups was similar. On the 90^th^ and the 120^th^ days, the rats in the sham group had normal development of the seminiferous tubules and the interstitial tissue (Figure 6). In both ligation groups, the rat testes were smaller and softer than that of the sham group. In the rat in the ligated group, the diameters of the seminiferous tubules and the thickness of the germinal epithelium were significantly decreased compared to the sham group (Table I). In the ligation group on the 90^th ^day, in 3 rats, and on the 120^th^ day in one rat, severe degeneration was seen in most seminiferous tubules with a normal view in a few tubules (Figure 7). When the tubules with a normal view were examined carefully, a decrease in the number of spermium in the tubule lumen was present. 

**Figure 8:**
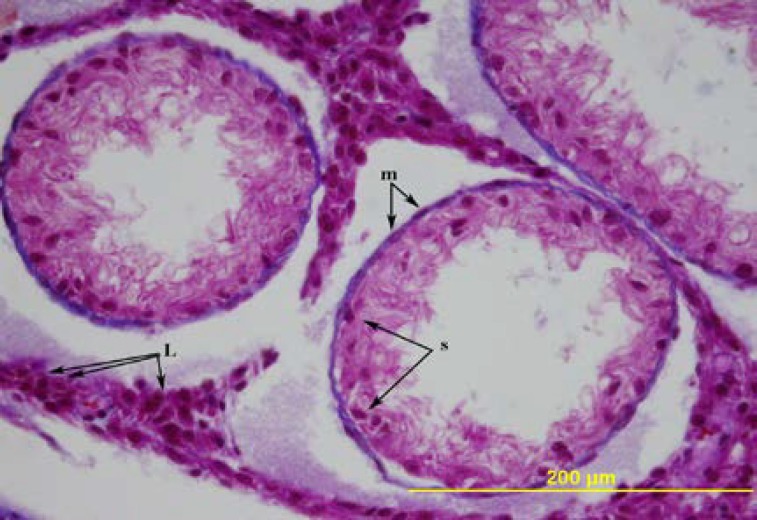
The testis of 120-day-old ligation group. m= peritubular myoid cell, s= Sertoli cell, L= Leydig cell. Triple stain X 400

**Figure 9:**
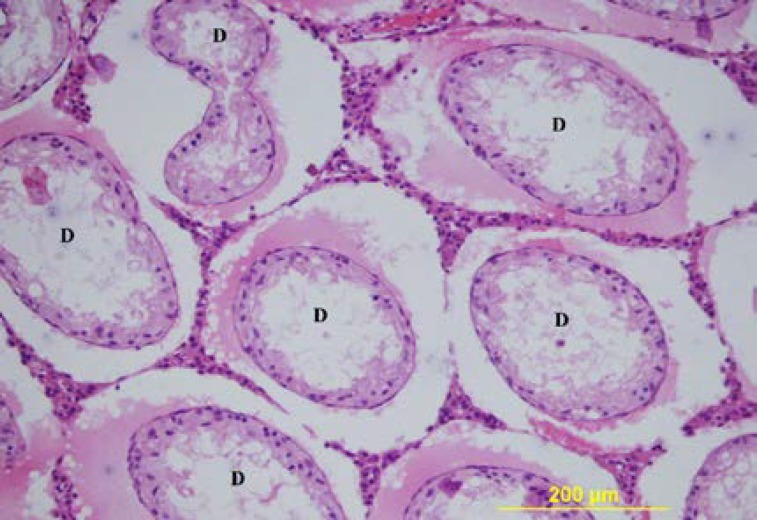
The testis of 120-day-old ligation group. D= degenerated tubules. Hematoxylin and eosin X 200

In one of the three rats on the 90^th^ day, in some of the seminiferous tubules there were multinucleate cells and round spermatids with similar features as the ligation group on the 56^th^ day. In the contralateral rat testes in these groups, all the seminiferous tubules were degenerated (Figure 8, 9). The degenerative changes in the in the seminiferous tubules of the rats in the ligation group on the 90^th^ and the 120^th^ days were similar. The diameters of the degenerated tubules and the thickness of the germinal epithelium were significantly decreased and the lumen of the tubuli was widened. In some of the tubuli, together with the Sertoli cells, there were spermatogonia, primary spermatocytes, and rarely spermatogenic cells like spermatids. In most of the tubuli, the seminiferous epithelium consisted of only Sertoli cells. 

No pathological condition was detected in the interstitial tissue of the both ligation groups. The number and the morphology of the Leydig cells were normal. The basal membranes of the degenerated tubuli seemed to stain darker by triple staining (Figure 8). The data concerning the average diameters of the seminiferous tubules and the average thickness of the germinal epithelium of the rat testes according to the days and the groups, and the number of observed degenerations are summarized in Table I.

**Table I:** In the ligature and control group rats the average seminiferous tubule's diameter and germinal epithelium thickness and the number of cases that observed degeneration

**Results**	**21 days**		**35 days**		**56 days**		**90 days**		**120 days**	
	**Sham**	**Ligation**	**Sham**	**Ligation**	**Sham**	**Ligation**	**Sham**	**Ligation**	**Sham**	**Ligation**
Mean seminiferous tubule diameter	120	118	195	198	273	297	317	211	320	220
Mean germinal epithelium thickness	41.5	43	71	71.5	87.5	73	89.5	35.5	93.5	35
Degeneration	-	-	-	-	-	1/5*	-	5/5	-	5/5

* : one of the five rats

## Discussion

In present study, no differences were encountered histologically and morphologically among the ligation group on the 21^st^ and the 35^th^ days, and in the sham group. These findings are in concordance with that of Flickinger *et al* (1). In all of the rats in the ligation group on the 56^th^ day, the diameter and the width of the lumen of the seminiferious tubules increased compared to the sham group. This increase is thought to arise from the accumulation of the tubular fluid and the sperm in this fluid, a result of the obstruction. 

This idea can explain the morphological differences between the ligation group and the sham group on the 56^th^ day. In rats in the ligation group, on the 90^th^ and the 120^th^ days, the diameter of the seminiferious tubules and the thickness of the germinal epithelium were significantly decreased compared to the sham group. This result explains the morphological differences between the ligation group and the sham group. The mentioned findings are similar to that of the available such as an increase in the diameter of the seminiferious tubules and the width of the lumen right after the obstruction, and in case of a long-term obstruction, this increase is followed by atrophic changes as a decrease in the thickness of the seminiferious epithelium and the tubulus diameter literature (1, 3, 13-15). 

The degenerative changes in the ligation group on the 90^th^ and the 120^th^ days are, in general, similar to that of Flickinger *et al* (1). However, in the rats of the ligation group the seminiferious tubules were not completely degenerated. In rats in the ligation group, on the 56^th^ and the 90^th^ days, the multinucleate cells seen in some of the degenerated seminiferious tubules were defined as multinucleate spermatids by Hess *et al* (16). 

These cells were interpreted as the most characteristic feature of the degeneration in the seminiferious tubules and an indicator of stress on the seminiferious epithelium (1, 9). In the seminiferious tubules, such entities are seen in different situations such as vasectomies ligation of the corpus epididymis and 1.3-Dinitrobenzene administration to the rats (1, 6, 9, 16). In the present study, different from the literature, multinucleate spermatids were also seen in the rats in the ligation group on the 90^th^ day (1). 

The other observations of the degenerative changes in the rats in the ligation groups, such as the changes in the diameter of the seminiferious tubules, the decrease in the thickness of the seminiferious epithelium and absence of the spermatogenic cells are in concordance with the findings in the literatures (1, 4, 6, 9, 10, 13-15). The basal membranes of the degenerated tubules were stained darker by triple staining. This was interpreted as an indicator of basal membrane thickening. This finding is similar to that of Jarow *et al* and Aktuğ *et al* (7, 13). Different from Aktuğ *et al* in this study, we have not observed any lymphocyte and macrophage infiltration to the seminiferious tubules and granuloma formation, and degenerative changes in the Leydig cells in the interstitial tissue (13).

There are various opinions about the etiology of the post-obstructive testicular damage. Vare and Bansal have claimed that the blood flow in the testes stands still due to the increase in the intratubular pressure, which leads to a decrease in the oxygenation of the tissues resulting in damage in the germinal epithelium, which is very sensitive to hypoxia (14). Pilsworth *et al* have mentioned the effect of the accumulation of the metabolites, whereas Soler *et al* have claimed that the endocrine factors secreted by the epididymis could have an effect in the nutrition of the testes (10, 11). 

Aktuğ *et al* reported that the damage in the integrity of the basal membrane has a key role whereas Flickinger *et al* claimed the increase of the anti-sperm antibodies to be the reason of the post-obstructive testicular damage (13, 17). In various animals, it has been shown that the rhythmic contraction and the relaxation of the small arteries and the arterioles in different tissues lead to changes in blood flow. This phenomenon, called vasomotion, is thought to regulate the nutrition of the tissues and the interstitial fluid exchange. Although there is no vasomotion in the prepubertal testes, it is observed in adult rats. Vasomotion in the testis is inhibited by hypoxia, cryptorchidism, varicocele and locally synthesized vasoconstrictors. The impairment of the vasomotion is followed by spermatogenic impairments (18). 

As mentioned in the literatures (likely right after the obstruction, because of the accumulation of the seminiferious fluid and spermium, the intratubular pressure will increase, and this condition, which leads to a decrease in the blood flow, will cause an accumulation of the metabolites and hypoxia and the impairment of the vasomotion (10, 11, 13, 14, 18). Due to the combined effects of the above-mentioned factors, the degenerative changes in the basal membrane and the germinal epithelium take place. 

Even after normalization of the intratubular pressure after cessation of the obstruction, the degenerative changes in the structure of the basal membrane and the impairment of the vasomotion can be permanent. This can explain the permanent character of the degenerative changes in the seminiferious epithelium, which is seen after the vaso-vasotomy, which is performed one month after the vasectomy, as described by Flickinger *et al *(6). Furthermore, the ligation of the corpus of the epididymis leads to more profuse and severe damage in the testes compared to the vasectomy. This result is supports the idea that endocrine factors secreted from the epididymis can be effective on the nurturing of the testis.

## Footnote

***This article extracted from Ph.D. thesis. (Fatih Mehmet Gur)***
